# Perceptions of procedural justice and coercion among forensic psychiatric patients: a study protocol for a prospective, mixed-methods investigation

**DOI:** 10.1186/s12888-020-02629-6

**Published:** 2020-05-13

**Authors:** Alexander I. F. Simpson, Irene Boldt, Stephanie Penney, Roland Jones, Sean Kidd, Arash Nakhost, Treena Wilkie

**Affiliations:** 1 Complex Care and Recovery Program, Centre for Addiction and Mental Health, 1001 Queen Street West, Toronto, Ontario M6J 1H4 Canada; 2 Department of Psychiatry, University of Toronto, Toronto, Canada; 3 Lawrence S. Bloomberg Faculty of Nursing, University of Toronto, Toronto, Canada; 4 Community Mental Health Services, St. Michael’s Hospital, 30 Bond St, Toronto, Ontario M5B 1W8 Canada

**Keywords:** Procedural justice, Coercion, Recovery, Risk assessment, Forensic mental health, Violence, Protective factors, Recidivism

## Abstract

**Background:**

The risk and recovery paradigms are the dominant frameworks informing forensic mental health services and have been the focus of increasing research interest. Despite this, there are significant gaps in our understanding of the nature of mental health recovery in forensic settings (i.e., ‘secure recovery’), and specifically, the key elements of recovery as perceived by forensic patients and their treatment providers. Importantly, we know little about how patients perceive the forensic mental health system, to what extent they see it as fair and legitimate, and how these perceptions impact upon treatment engagement, risk for adversity, and progress in recovery.

**Methods:**

In this prospective, mixed-methods study, we investigate patient perceptions of procedural justice and coercion within the context of the forensic mental health system in Ontario, Canada (final *N* = 120 forensic patients and their primary care providers). We elicit patient self-assessments of risk and progress in recovery, and assess the degree of concordance with clinician-rated estimates of these constructs. Both qualitative and quantitative methods are used to assess the degree to which patient perceptions of coercion, fairness and legitimacy impact upon their level of treatment engagement, risk for adversity and progress in recovery. A prospective, two-year follow-up will investigate the impact of patient and clinician perspectives on outcomes in the domains of forensic hospital readmission, criminal reoffending, and rate of progress through the forensic system.

**Discussion:**

Results from this mixed-methods study will yield a rich and detailed account of patient perceptions of the forensic mental health system, and specifically whether perceptions of procedural fairness, justice and legitimacy, as well as perceived coercion, systematically influence patients’ risk for adversity, their ability to progress in their recovery, and ultimately, advance through the forensic system towards successful community living. Findings will provide conceptual clarity to the key elements of secure recovery, and illuminate areas of similarity and divergence with respect to how patients and clinicians assess risk and recovery needs. In doing so, knowledge from this study will provide a deep understanding of factors that promote patient safety and recovery, and provide a foundation for optimizing the forensic mental health system to improve patient outcomes.

## Background

Forensic patients are most commonly defined internationally as persons with a serious mental disorder who have come in contact with or may come in contact with the criminal justice system. In Canada, the forensic patient designation is usually confined to persons who have been found by a court to be either unfit to stand trial (UST) or not responsible for a criminal offense on account of mental disorder (NCRMD). The care and recovery framework for such patients – approximately 4500 individuals in Canada – is overseen by provincial Review Boards constituted under the Canadian Criminal Code (CCC s. 672.34). Review Boards are responsible for annually reviewing the status of every person under their jurisdiction and making ultimate decisions regarding the least restrictive placement of the individual (i.e., continued detention, conditional or absolute discharge) having regard to public safety. Judgments regarding progression through the forensic system (e.g., to conditional and absolute discharge) are predicated on decisions of risk – that is, whether the person continues to pose a significant threat to the community, defined as *a real risk of physical or psychological harm to members of the public that is serious in the sense of going beyond the merely trivial or annoying* (CCC s. 672.54; the conduct giving rise to the harm must also be criminal in nature).

Risk – both for physical and psychological harm – is therefore of central importance to the mental health provisions of the Criminal Code. It is the focal point of almost all decision-making in forensic mental health services, from treatment planning, to determinations of patient security level, provision of community access privileges, and decisions to conditionally and absolutely discharge patients from the system. Measures that assess empirically-supported risk factors for future violence and criminal offending are routinely used to evaluate forensic patients and make decisions about how best to meet their needs while also protecting the safety of the public.

Alongside measures of risk, it is increasingly acknowledged that forensic assessments need to incorporate recovery-oriented and strengths-based factors to accurately gage patients’ clinical and rehabilitative progress. Indeed, the focus on risk for adversity has seriously limited investigation of successful outcomes among forensic patients, and more broadly, of their recovery process and quality of life. This is a serious deficiency in forensic research and service provision, particularly as recovery-based philosophies, which promote patient voice and self-determination, have come to dominate expectations on mental health services in Canada and other countries [29]. As we have recently argued [40], recovery for forensic service users has the core elements of recovery for all persons with a serious mental illness but also offence specific themes, and themes derived from the nature of their detention and legal oversight. Little research has been performed to elucidate these themes.

Canadian forensic services are organized by province and vary from one province to another. In Ontario (the location of the current study), secure hospital services include one maximum security hospital and 10 regional forensic hospitals containing both medium and low security beds in varying proportions depending on the program. Generally, approximately half of all forensic patients in Ontario are inpatients and the other half under forensic outpatient care. Graduated hospital grounds and community access (in the form of progressing passes and privileges) is granted by the hospital within an envelope of available passes defined in the annual disposition issued by the provincial review board [41].

Of specific interest in this proposed study is whether patients consider their involvement in forensic mental health system as legitimate, whether they feel they are treated fairly, and whether these perceptions impact how they engage with treatment and risk management efforts, and ultimately, progress through the forensic system toward successful community living. Exploring patient perceptions of legitimacy, which are based on perceptions of procedural justice [46] and likely also affected by experiences of coercion arising from their involuntary status, is particularly relevant in the understanding how patients engage in recovery. As Livingston [24] has recently shown, forensic patients are acutely aware of the competing processes of the need to live a “compliant life” with the needs to develop personal independence. Others have similarly highlighted the tension – and often perceived incompatibility – between recovery principles and the nature of secure care (e.g., promoting autonomy and self-determination under conditions of legal coercion [17];). These themes of ambivalence, and the experience of the journey through secure care as being limiting, though necessary, for recovery have been described in the systematic review of forensic user experiences by Shepherd et al. [39] and the more recent work of Livingston [24], Aga et al. [2] and Tomlin et al. [45].

To explore these themes further, this study assesses patient perceptions of procedural justice and coercion within the Canadian forensic mental health system. Using quantitative and qualitative approaches, we investigate the degree to which patient perceptions within these domains impact upon their level of treatment engagement, risk for adversity (e.g., reoffending, readmission to secure care) and progress in recovery. Patient- and clinician-rated assessments of risk and recovery are also collected to inform what may be areas of agreement and divergence with respect to the estimation of risk and recovery needs. Finally, we prospectively examine how this constellation of variables impacts upon the actual likelihood of an adverse outcome and rate of progress through the forensic system.

### Risk assessment and its relationship with patient recovery

As principles of secure recovery have evolved over the past decade, so too have considerations of best-practices in violence risk assessment and management, and in particular, how to better align the practice of risk assessment with recovery principles. Traditionally, the literature has focused almost exclusively on the predictive efficacy of professional risk assessment schemes, with little attention paid to the ways in which patients perceive their own risk and how their perspectives can or should be woven into their assessments. Forensic patients report being minimally aware of the content of their risk assessments, and that they had been informed of the risk judgments rather than asked to contribute to the formulation [14]. Dixon [14] also found that the level of agreement between clinicians and patients was low; they had differing views about the reasons for their offending behavior and about appropriate risk management strategies. Other studies have similarly found poor concordance between patient- and clinician-rated assessments of risk and protective factors (e.g., [47]), although this concordance strengthens as patients progress in their recovery pathways.

Results suggesting suboptimal agreement between patient- and clinician-formulated risk is perhaps the expected outcome of a tradition of doing risk assessments “to” rather than “with”. In contrast, a collaborative approach to risk assessment would serve to better align this practice with a recovery framework, in part, by promoting patient voice, empowerment, and engagement with treatment providers [4]. Furthermore, a shared decision-making model for risk (e.g., [5, 8, 14, 44]) would increase the likelihood that resulting risk formulations and management plans are relevant and palatable to the patient, and encourage a deeper understanding and mastery over risk issues. This, in turn, would presumably be associated with fewer adverse outcomes as patients’ progress in their recovery. At present, however, the utility and reliability of patient self-assessments remains to be shown in an empirical manner.

Alongside the absence of patient voice and perspective, the sole focus on risk for adversity (i.e., violence and offending) has significantly curtailed knowledge regarding those variables which promote successful outcomes for forensic patients, whether in hospital or upon transition to the community. In the context of forensic assessments, the failure to consider protective or strengths-based factors can result in inaccurate, overly pessimistic predictions of risk [11]. Protective factors may simply be the absence of risk in a particular domain (e.g., no history of substance abuse), but also include positive personal characteristics (e.g., coping skills, employability), social factors (e.g., peer support, prosocial relationships), and factors related to treatment compliance and responsivity [11, 13]. At present, we know strikingly little about the prevalence of strengths-based factors and successful outcomes experienced by forensic patients as they recover and progress from inpatient forensic care to the community [24, 42].

### Perceived procedural justice and coercion

Procedural justice (PJ) relates to the fairness and transparency of process by which decisions are made. PJ theory posits that satisfaction with legal or clinical decisions is primarily determined by the quality of the procedural experience rather than the outcome of the decision. Decisions that are perceived as procedurally just involve respect and dignity, participation in the decision-making process, trust in the fairness of the process, and the absence of coercion.

Available evidence suggests that a fair and transparent legal process has beneficial effects on clinical outcomes. For instance, perceived PJ is found to be positively correlated with favorable attitudes toward recovery for individuals undergoing mental health court diversion [22]. In contrast, perceptions of coercion are inversely associated with perceptions of fairness and legitimacy with respect to decision-making in general psychiatric services [23, 27, 28, 36]. Involuntary treatment, which reduces patient autonomy and liberty, is associated with increased patient perceptions of coercion relative to voluntary patients, although voluntary patients also commonly experience coercion in relation to health care decisions [31, 36].

The related constructs of PJ and perceived coercion (PC) are particularly relevant to consider within the context of forensic mental health care, given the real limits that are imposed on patient autonomy and liberty in the name of public safety. To date, there have been a limited number of investigations of forensic patients’ experiences of PJ and PC (e.g., [15]), such that the role of PJ and PC on risk and recovery-based outcomes is largely unknown [7]. It is conceivable that perceptions of coercion and negative appraisals of procedural justice would adversely affect risk management and rehabilitative efforts, particularly given that PC is associated with increased levels of negative affect, notably anger and resentment [26, 30, 49], which are themselves risk factors for violence [16] and common motivating factors for absconding from forensic hospitals [48]. PC may also play a role in the high levels of poor treatment engagement observed among forensic patients [33]. Furthermore, a lack of choice and negative perceptions of treatment are key factors for poor engagement in both forensic and correctional populations [43].

In the general population, perceptions of the law’s fairness and legitimacy results in greater acceptance and compliance with legal rules and processes [46], and may promote the internalization of the value of rules and law [22]. By extension, forensic patients may be more engaged, compliant and responsive to treatment efforts if they perceive their involvement in the forensic system as procedurally just and legitimate.

## Methods/design

### Study aims and hypotheses

To date, there is little information about how forensic patients experience their care and supervision under the forensic mental health system, to what extent they perceive the system as fair and legitimate, and how these perceptions impact upon treatment engagement, risk, and progress in recovery. The principal aim of this study is to explore forensic patient perceptions of procedural justice and experiences of coercion, and to relate these perceptions to relevant clinical outcomes in the domains of treatment engagement, risk for adversity and progress in recovery. A secondary aim is to assess the degree of agreement between patient and clinician estimates of risk and progress in recovery, explore areas of relative concordance and divergence.

We hypothesize that (1) the experience of PJ will be positively associated with treatment engagement and progress in recovery, while (2) higher levels of PC will be inversely associated with these outcomes, and positively associated with measures of risk and adversity. Further, we hypothesize that (3) greater patient-clinician concordance with respect to estimates of risk and recovery will be associated with a reduced likelihood of hospital readmission, reoffending and prolonged tenure under the supervision of the forensic system. To isolate the effects of PJ and PC, hypotheses will be tested in models controlling for variables related to progress in recovery (namely, the presence of protective factors as measured by the SAPROF, described below) as well as variables related to the likelihood of adversity in the domains of hospital readmission, reoffending and prolonged forensic system tenure (i.e., risk factors as measured by the HCR-20^V3^, described below). Patients’ current degree of liberty/restriction (i.e., current level of security, current passes and privileges granted) will also be accounted for.

### Design

**Fig. 1:**
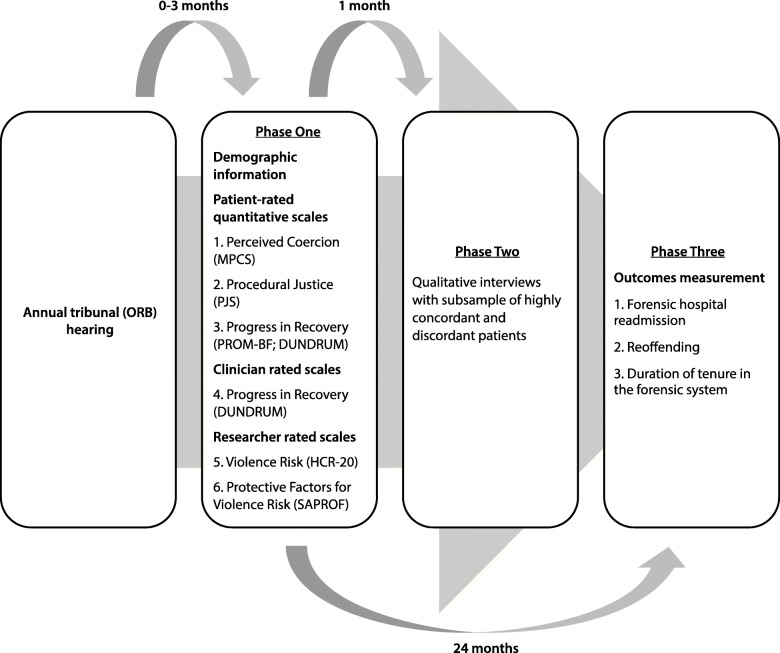
Study Design

See Fig. 1 for a depiction of the study design. This study will use a sequential mixed-methods design with a prospective component. The mixed-methods portion of the study will be carried out in two phases; in phase one the quantitative data will be collected, and in phase two the qualitative data will be gathered. The prospective portion of the study will be phase three.

As depicted in Fig. 1, a mixed-methods sequential design is typically characterized by the collection and analysis of quantitative and then qualitative data in two consecutive phases within one study. A final phase entails the integration or linking of data from the two separate phases of data collection. A central advantage of a mixed-methods approach is that it can draw upon the respective strengths of both quantitative and qualitative research methodologies, and that findings from each method may be integrated, either concurrently or sequentially, in order to more fulsomely and robustly address the overarching research objectives [20].

As applied to this study, the quantitative data are collected and analyzed prior to the qualitative data, while the latter are used primarily to help explain and elaborate on the quantitative results obtained. Quantitative and qualitative findings will be integrated with the use of a joint display, which allows data to be visually brought together [18]. Sample quotes from the qualitative interviews will be compared and contrasted with findings generated from the quantitative analyses. Areas of relative convergence and divergence between the qualitative and quantitative results will be distilled and interpreted in the final analysis of results. Relatively greater weight will be afforded to the quantitative results in the final interpretation and discussion of results, given that these data are drawn from the total sample, rather than a smaller subsample as is be the case for the qualitative findings.

### Setting and participants

Participants will be 120 patients recruited from adult inpatient and outpatient forensic services within a large urban psychiatric hospital in Ontario, encompassing 180 forensic inpatient beds and approximately 240 outpatients. All participants will have been adjudicated as not responsible for a criminal offense on account of mental disorder (NCRMD) and will be under the supervision of the Ontario Review Board (ORB). Quota sampling will be used to select a sample of inpatients (*n* = 60) and outpatients (*n* = 60) who have had their annual review by the ORB within the last 3 months. Given our prior work detailing the sociodemographic and clinical characteristics of all forensic patients in Ontario ([35]; described below), we will be able to ascertain whether the current sample is representative of the larger patient population in the province.

Anticipated sample characteristics may be gleaned from our prior population-based study of all Ontario forensic admissions from 1987 to 2012 resulting in a disposition of NCRMD (*N* = 2533 [35];). The majority were male (86%), had less than a high school education (48%), and were never married (58%). Most (82%) had committed at least one violent offense as part of their admitting offense to the hospital. Eighty-two percent had a primary psychotic disorder (schizophrenia in 57%, other psychoses in 25%), and 49% were diagnosed with a comorbid substance use disorder. The average length of time spent in the forensic system was over 7 years (M = 7.8 [SD = 6.0]).

Power calculations for each of the study’s hypotheses is somewhat complicated by the lack of prior effect size estimates available in the literature. Nevertheless, an *N* = 120 is in the range of previous studies on PC and PJ (e.g., [31, 36]), as well as investigations of the DUNDRUM-3 and DUNDRUM-4 [9, 10, 32]. If moderate effect sizes (i.e., *d* = 0.5–0.8) are anticipated with respect to the associations between PJ, PC and treatment engagement / progress in recovery, a sample size of 120 (allowing for a modest amount of attrition) yields a power of at least 95% with alpha = 0.05 to detect an association between these variables.

#### Recruitment

Subjects will be recruited within 3 months of their annual ORB hearing. Potential participants will be referred to the study by their primary clinician if they deem that the patient has the capacity to consent and participate in the study. The approach to participate is by research staff, not by members of the treatment team. The recruiting individual will provide interested participants with a consent form, and will attain written consent from individuals wishing to participate. Prior to signing the consent form participants will have the chance to ask and have answered any questions they might have. Additionally, in order to determine whether the participant understands the purpose of the study and their role within the study, patients will be asked the following questions; 1) In your own words, please describe what the study is about; 2) What are some benefits of participating in this study?; 3) What are some risks of participating in this study?; and 4) Will your participation influence decisions made by the ORB? These questions are based on the factors to consider for obtaining consent, as outlined by the Personal Health Information Protection Act (PHIPA, 2004). Potential participants will be approached until we achieve our sample size of 60 in each of the inpatient and outpatient samples.

### Measures

The following quantitative measures will be administered during phase one of the study to assess the specified constructs of interest.

#### Perceived coercion (PC)

The MacArthur Perceived Coercion Scale (MPCS [19];) is a widely-used survey designed to assess perceptions of coercion in psychiatric patients. Our version adapts the questions for use in both inpatient and outpatient forensic settings and consists of 8 items, each focusing on one aspect of PC: influence, control, choice, freedom, persuasion, inducements, threats, and force (e.g., How much influence do you have on your personal and legal/health situation? How much is your personal and legal/health situation based on your choice? Did you feel as though anyone tried to force you into accepting your personal and legal/health situation?). Participants’ responses will be recorded using a 7-point Likert scale where higher values indicate greater PC.

#### Procedural justice (PJ)

The PJ Scale (PJS [23];) is used to measure patients’ perceptions of PJ surrounding their ORB hearing and the decisions made regarding their ongoing treatment and supervision within the forensic mental health system. Our adapted PJS consists of 9 items, each focusing on one dimension of PJ: motivation, respect, validation, fairness, information, voice, deception, interest, and satisfaction. Questions appearing on the PJS will be asked separately for the patient’s healthcare team, psychiatrist, lawyer, and members of ORB, for a total of 36 questions. For example, in the domain of respect, the same question will be asked for each of the four groups listed above (i.e., how much respect did your healthcare team/psychiatrist/lawyer/ORB) treat you with?).

The above two scales have been used previously in forensic settings [25] and have shown results consistent with their use in general mental health settings. They have also been adapted for community mental health settings [26] and are the most commonly used measures of coercion in compulsory community treatment care studies (e.g., [36]).

#### Treatment engagement / Progress in recovery

The Programme Completion and Recovery scales of the Dangerousness, Understanding, Recovery, and Urgency Manual (DUNDRUM [21];) are designed to assess patient engagement and progress in treatment, as well as progress in recovery. The Programme Completion scale (7 items) relates to progress and engagement in key “pillars of care”: physical health, mental health, drugs and alcohol, problem behaviours, self-care and activities of daily living, education, occupation and creativity, and family and social networks. The Recovery scale (6 items) includes items assessing stability, insight, rapport / working alliance, leave, dynamic violence risk, and victim sensitivities.

The DUNDRUM scales all have parallel patient- and staff-rated versions, enabling a comparison between these user groups on estimates of progress in treatment and recovery, and related areas of unmet treatment need/risk. The patient- and clinician-rated versions of the scales have excellent internal consistency, are correlated with each other (Spearman’s rho = 0.57 [Programme Completion], 0.71 [Recovery]), and predict inpatient violence, inpatient self-harm, conditional discharges to the community, and progress along the recovery pathway [1, 9, 10].

The Personal Recovery Outcome Measure-Brief Form (PROM-BF [3];) is a 10-item measure designed to assess personal recovery of people with mental illness living in the community. The measure was developed by drawing upon existing measures of recovery and using an iterative process of qualitative and quantitative testing guided by Rasch Measurement Theory. Each item (e.g., I am motivated to keep myself well; I can identify the early warnings signs of becoming sick; I accomplish the goals I set out for myself) has a 5 point response scale with 0 = *None of the time* and 4 = *100% of the time*. Although the scale was originally developed and validated on community-dwelling individuals with mental illness, we administer the PROM-BF to both inpatients and outpatients in this study as it was deemed that all items are relevant and appropriately phrased for both patient groups.

#### Violence risk

Risk status will be measured with the Historical, Clinical and Risk Management-20, Version 3 (HCR-20^V3^ [16];), a widely used and extensively validated structured professional tool to assess violence risk. It consists of 10 risk factors relating to historical variables (e.g., previous violence, past problems with substance use or employment, trauma history), 5 items describing the patient’s current clinical concerns (e.g., insight, active symptoms of major mental illness, current treatment compliance), and 5 items describing areas for future risk management (e.g., future plans for housing or employment, presence of social supports). Each item may be scored on a three-point scale as 0 (not present), 1 (possibly or partially present), or 2 (definitely present). A final summary risk rating is then presented as low, moderate, or high.

#### Protective factors for violence risk

The Structured Assessment of Protective Factors for violence risk (SAPROF [11];) is a structured professional judgment tool consisting of 17 protective factors which are rated on a three-point scale (0–2) reflecting the degree to which the protective factor is judged to be present. Items are organized within three scales: Internal factors (e.g., intelligence, empathy, self-control), Motivational factors (e.g., motivation for treatment, life goals), and External factors (e.g., social network, living circumstances). After rating the protective factors, an overall protection judgement (low, moderate, high) is made regarding the level of protection from relapse. A total sum score of the 17 SAPROF item scores can also be calculated. Recent research has demonstrated the predictive validity of the SAPROF in forensic psychiatric settings in relation to reduced rates of adverse clinical outcomes (e.g., inpatient violence, self-harm [1, 12];).

### Procedure

#### Phase one

First, a review of patients’ health record information is conducted to gather sociodemographic information (e.g., age, sex, primary diagnosis) and rate the risk and protective factors appearing on the HCR-20^V3^ and SAPROF (see Measures section). Once the file review is complete, the same member of the research team schedules an interview with the participant, at which time the quantitative self-report questionnaires assessing procedural justice, perceived coercion, and recovery are administered. At present, 28 patients have completed phase one of the study. This recruitment occurred over a three-month period. We anticipate it will take 1 year to recruit the full sample for the study.

#### Phase two

In the second stage, a subset of approximately 20 participants who consent to being re-contacted are purposively sampled for a follow-up one-to-one qualitative interview performed within 4 weeks of their first appointment. Specifically, those who are highly concordant (~ 10 patients) and discordant (~ 10 patients) with their primary clinician’s ratings on the DUNDRUM Programme Completion and Recovery scales are sampled, creating two groups. The qualitative research paradigm would suggest that this size of sample per group is adequate in the context of the methods proposed in this study [34, 38].

Mean concordance scores across the DUNDRUM Programme Completion and Recovery scales are used to guide participant selection in phase two. Highly discordant participants are defined as those whose average concordance score on either or both the DUNDRUM Programme Completion and Recovery scales is greater than or equal to one standard deviation above the overall mean (M = 1.1, SD = 0.8) concordance score across the two scales found in Davoren et al. [10] (lower values = greater concordance). Conversely, highly concordant patients are defined as those whose average concordance score on both of the two scales is less than or equal to one standard deviation below this published norm.

To get a representative sample of the forensic population at the hospital, our purposive sampling strategy also considers the age, sex, status (outpatient, inpatient), and security level (type of inpatient unit – minimum or medium) when selecting participants to conduct follow-up qualitative interviews.

#### Qualitative interview structure

Semi-structured interviews with a selection of the highly concordant/discordant patients last approximately 1 h. Interviews consist of open-ended questions that elicit and probe participants’ perspectives and experiences regarding their time under the ORB and their current clinical care. Specifically, patients are asked about: (1) their experiences of being under the ORB; (2) whether they perceive their being under the ORB as legitimate (e.g., fair, reasonable, right) both at present and when they were first under the ORB; (3) their perception of their own risk, as well as if and how this relates to their being under the ORB; (4) their perceptions of their own recovery, as well as if and how this relates to their being under the ORB; and, (5) their understanding of the relationship between risk and recovery.

#### Phase three

Outcome data in the domains of forensic hospital readmission, criminal reoffending, and duration of tenure within the forensic system is collected at 24 months following the first interview (phase one). Rates of hospital readmission (for outpatients or patients achieving discharge during the study window) are collected from the health record. Rates of reoffending are tracked via records from the Canadian Police Information Centre (CPIC). Lastly, length of stay within the forensic system is calculated by subtracting the date of admission from three separate dates representing recovery-oriented milestones in the trajectory of forensic care: (1) initial discharge from hospital into the community; (2) conditional discharge from the ORB; and (3) absolute discharge from the ORB (i.e., complete cessation of forensic patient status).

### Statistical analysis

All analyses of the data collected in phase one will be carried out using R with a conventional two-sided *p* < 0.05. Bivariate correlations will first be computed to examine the associations between PJ, PC and measures of treatment engagement and recovery (i.e., DUNDRUM scales). Then, scores on our measures of treatment engagement and recovery will be regressed on scores from the PJ and PC scales while controlling for scores on the SAPROF and HCR-20^V3^. A series of linear regressions will be conducted to examine the predictive efficacy of patient and clinician ratings on prospectively-measured, dichotomously-coded outcomes in the domains of hospital readmission and reoffending. Length of stay (inpatient) and total duration of time spent under forensic supervision will be included as covariates in these models. Survival analysis will be used to model the duration of time elapsing from the point of admission to the gradual points of discharge from the forensic mental health system (i.e., community living, conditional and absolute discharge).

Patient-clinician agreement on the DUNDRUM Programme Completion and Recovery scales will be assessed by examining the bivariate correlations between patient- and clinician-generated scores. Unstandardized difference scores will be calculated in order to examine the magnitude and direction of discrepancies between patient and clinician ratings. The unstandardized difference score is obtained by simply subtracting the raw score of one informant from a second informant’s raw score. Lastly, difference scores will be entered into a regression model to investigate whether the degree of patient-clinician concordance is associated the likelihood of adversity at the 24-month follow-up.

#### Qualitative analysis

All interview data gathered in stage two, with the consent of participants, is audio-recorded and transcribed verbatim. We will be guided by Braun and Clarke’s [6] method of thematic analysis, involving six discrete steps. First, *familiarizing yourself with your data* requires repeated engagements with the data and noting preliminary ideas and areas of analytic interest. Second, *generating initial codes* involves producing preliminary codes from the entire data set. Third, *searching for themes* involves organizing the codified data into potential themes and subthemes. Fourth, *reviewing themes* requires considering, and revising as needed, the internal coherence of the codes in each theme as well as the overall coherence of the identified themes in relation to the entirety of the data. Fifth, *defining and naming themes* involves refining, defining, and naming each theme. Finally, *producing the report*, the last level of analysis, involves selecting the most suitable way to present the data and explicitly linking it to the research questions and the extant literature.

The qualitative data analysis will be conducted with the assistance of NVivo software. For quality control and consistency, 10–15% of the transcripts will be double-coded by a second rater who will be blinded to the coding of the primary rater. Additionally, initial themes, findings and concerns will be brought to the research team for discussion. Preliminary analyses will be performed concurrently with data collection, and emerging themes may inform the focus of subsequent interviews as well as the point at which thematic saturation has been reached.

As noted above (section 2.2), quantitative and qualitative findings will be visually integrated in the final analysis and sample quotes from the qualitative interviews will be compared and contrasted with findings generated from the quantitative analyses. Areas of relative convergence and divergence between the qualitative and quantitative results will be distilled and interpreted in the final analysis of results. The qualitative data and their analysis is anticipated to refine and explain the quantitative results by exploring participants’ views in more depth, and potentially uncovering new conceptual domains of risk and recovery not covered by the quantitative scales employed.

### Ethical approval

Ethical approval has been gained from the Centre for Addiction and Mental Health Research Ethics Board (approval number CAMH REB 046–2017). It is funded through a peer audited Centre for Addiction and Mental Health Foundation Grant from the Grey Foundation (Grant number K Grey Foundation RE092 Law and Mental Health Program).

## Discussion

This study will be one of the first to comprehensively investigate patient perceptions of care under the forensic mental health system, and specifically, to explore the impact of these perceptions on relevant clinical outcomes in the domains of treatment engagement, risk for adversity, and progress in recovery. Our focus on perceptions of procedural justice and coercion is particularly germane given the major loss of liberty associated with secure care and the length of inpatient forensic hospitalizations. By combining quantitative and qualitative methodological approaches, and both patient and care provider viewpoints, the data generated from this study will yield a nuanced understanding of the factors that promote or hinder successful outcomes and recovery efforts among forensic patients.

The investigation of patient and clinician agreement with respect to progress in treatment and recovery is particularly novel, as only a few studies to date have looked at the degree of agreement and its impact on progress and outcomes. Shared decision making processes are lacking in forensic mental health [37] and the DUNDRUM is one of the few measures that will allow us to explore these dimensions. Finally, given that the quantitative exploration of this constellation of concepts is novel, the addition of a qualitative approach is crucial. This will promote a richer understanding of patient experiences, thereby allowing us to critically reconsider the risk and recovery frameworks employed in forensic interventions and ORB processes.

## Data Availability

The data that support the findings of this study will be available on request from the corresponding author once the study is published. The data are not publicly available due to the sensitivity of the subject and participants.

## Abbreviations

DUNDRUM: Dangerousness Understanding, Recovery and Urgency Manual
HCR-20^V3^: Historical Clinical Risk Management-20, Version 3
LRS: Level of Restrictions Scale
MPCS: MacArthur Perceived Coercion Scale
NCRMD: Not Criminally Responsible on account of Mental Disorder
ORB: Ontario Review Board
PC: Perceived coercion
PJ: Procedural justice
PJS: Procedural Justice Scale
PROM-BF: Personal Recovery Outcome Measure-Brief Form
SAPROF: Structured Assessment of Protective Factors for Violence Risk
